# Infants’ Diapers

**Published:** 1870-12

**Authors:** 


### INFANTS' DIAPERS.

We have recently seen an article, called the Eureka Diaper, which, being waterproof, protects the clothing and bedding. It holds the linen diaper in place, and, when on, looks like a very neat-fitting pair of drawers. It is rapidly coming into general use, for mothers who have tried it have in gratitude volunteered their written statements that it is a perfect success, and prominent physicians have expressed in writings which we have seen, their high appreciation of the article. To be found at the principal dry goods stores throughout the country.

